# CD8 T cells compensate for impaired humoral immunity in COVID-19 patients with hematologic cancer

**DOI:** 10.21203/rs.3.rs-162289/v1

**Published:** 2021-02-02

**Authors:** Erin M. Bange, Nicholas A. Han, Paul Wileyto, Justin Y. Kim, Sigrid Gouma, James Robinson, Allison R. Greenplate, Florence Porterfield, Olutosin Owoyemi, Karan Naik, Cathy Zheng, Michael Galantino, Ariel R. Weisman, Caroline A.G. Ittner, Emily M. Kugler, Amy E. Baxter, Olutwatosin Oniyide, Roseline S. Agyekum, Thomas G. Dunn, Tiffanie K. Jones, Heather M. Giannini, Madison E. Weirick, Christopher M. McAllister, N. Esther Babady, Anita Kumar, Adam J Widman, Susan DeWolf, Sawsan R Boutemine, Charlotte Roberts, Krista R Budzik, Susan Tollett, Carla Wright, Tara Perloff, Lova Sun, Divij Mathew, Josephine R. Giles, Derek A. Oldridge, Jennifer E. Wu, Cécile Alanio, Sharon Adamski, Alfred L. Garfall, Laura Vella, Samuel J. Kerr, Justine V. Cohen, Randall A. Oyer, Ryan Massa, Ivan P. Maillard, The UPenn COVID Processing Unit, Kara N. Maxwell, John P. Reilly, Peter G. Maslak, Robert H. Vonderheide, Jedd D. Wolchok, Scott E. Hensley, E. John Wherry, Nuala Meyer, Angela M. DeMichele, Santosha A. Vardhana, Ronac Mamtani, Alexander C. Huang

**Affiliations:** 1 Division of Hematology/Oncology, Department of Medicine, Perelman School of Medicine, University of Pennsylvania; 2 Abramson Cancer Center, University of Pennsylvania; 3 Institute for Immunology, Perelman School of Medicine, University of Pennsylvania; 4 Human Oncology and Pathogenesis Program, Memorial Sloan Kettering Cancer Center; 5 Department of Medicine, Memorial Sloan Kettering Cancer Center; 6 Department of Laboratory Medicine, Memorial Sloan Kettering Cancer Center; 7 Department of Biostatistics, Epidemiology, and Informatics, Perelman School of Medicine, University of Pennsylvania; 8 Division of Hematology/Oncology, Department of Medicine, Perelman School of Medicine, Presbyterian Hospital; 9 Division of Pulmonary and Critical Care, Department of Medicine, Perelman School of Medicine, University of Pennsylvania; 10 Division of Hematology/Oncology, Department of Medicine, Perelman School of Medicine, Presbyterian Hospital; 11 Division of Hematology/Oncology, Department of Medicine, Perelman School of Medicine, Pennsylvania Hospital; 12 Division of Hematology/Oncology, Department of Medicine, Lancaster General Hospital; 13 Department of Systems Pharmacology and Translational Therapeutics, Perelman School of Medicine, University of Pennsylvania; 14 Department of Pathology and Laboratory Medicine, Perelman School of Medicine, University of Pennsylvania; 15 Parker Institute for Cancer Immunotherapy; 16 Department of Microbiology, Perelman School of Medicine, University of Pennsylvania; 17 Department of Pediatrics, Perelman School of Medicine, Children’s Hospital of Philadelphia

## Abstract

Cancer patients have increased morbidity and mortality from Coronavirus Disease 2019 (COVID-19), but the underlying immune mechanisms are unknown. In a cohort of 100 cancer patients hospitalized for COVID-19 at the University of Pennsylvania Health System, we found that patients with hematologic cancers had a significantly higher mortality relative to patients with solid cancers after accounting for confounders including ECOG performance status and active cancer status. We performed flow cytometric and serologic analyses of 106 cancer patients and 113 non-cancer controls from two additional cohorts at Penn and Memorial Sloan Kettering Cancer Center. Patients with solid cancers exhibited an immune phenotype similar to non-cancer patients during acute COVID-19 whereas patients with hematologic cancers had significant impairment of B cells and SARS-CoV-2-specific antibody responses. High dimensional analysis of flow cytometric data revealed 5 distinct immune phenotypes. An immune phenotype characterized by CD8 T cell depletion was associated with a high viral load and the highest mortality of 71%, among all cancer patients. In contrast, despite impaired B cell responses, patients with hematologic cancers and preserved CD8 T cells had a lower viral load and mortality. These data highlight the importance of CD8 T cells in acute COVID-19, particularly in the setting of impaired humoral immunity. Further, depletion of B cells with anti-CD20 therapy resulted in almost complete abrogation of SARS-CoV-2-specific IgG and IgM antibodies, but was not associated with increased mortality compared to other hematologic cancers, when adequate CD8 T cells were present. Finally, higher CD8 T cell counts were associated with improved overall survival in patients with hematologic cancers. Thus, CD8 T cells likely compensate for deficient humoral immunity and influence clinical recovery of COVID-19. These observations have important implications for cancer and COVID-19-directed treatments, immunosuppressive therapies, and for understanding the role of B and T cells in acute COVID-19.

Severe illness affects up to 20% of those hospitalized with Coronavirus Disease 2019 (COVID-19)^1^ and is manifested by acute respiratory distress syndrome (ARDS), multi-organ failure, and/or death^2^. Severe disease has been linked to immune dysregulation, including deficiency in the production of type I and type III interferons^3–5^, marked lymphopenia^6–10^, and a paradoxical increase in pro-inflammatory cytokines, such as TNFα, IL-1β, and IL-6^3, 6, 11–15^. In addition, alteration of the lymphocyte compartments has been reported during COVID-19 with increases in activated CD4 and CD8 T cells^15–18^, skewing of CD8 T cells towards effector^16, 17^ and exhausted phenotypes^18^, and increased differentiation of CD4 T cells towards the Th17 lineage^17, 19^. Despite these marked alterations in their T cell compartment, COVID-19 patients have robust plasmablast responses^15, 20^, and the majority of patients generate IgM and IgG antibodies to SARS-CoV-2 over the course of disease^20–22^. More recently, integrated and multi-omic analyses have highlighted the tremendous heterogeneity of the human immune response to SARS-CoV-2, with distinct immunophenotypes that are associated with COVID-19 disease severity and disease trajectory^5, 11, 12, 15, 16^. Understanding how clinical features, particularly patient comorbidity, impact host immune responses to SARS-CoV-2 will elucidate determinants of immunotype and disease severity.

Cancer patients have an increased risk of severe illness from COVID-19^23–26^ with an estimated case fatality rate of 25%^27^ compared to 2.7% in the general population^28^. Importantly, cancer is a heterogeneous disease with even higher mortality rates reported for patients with particular subtypes of cancer. For example, several cohort and registry studies have demonstrated particularly poor outcomes among patients with hematologic cancers, with mortality rates as high as 55%^23, 26, 29–37^. However, it remains unknown whether the increased mortality by cancer subtype is independent of the confounding effects of other prognostic factors such as Eastern Cooperative Oncology Group (ECOG) performance status, active cancer status, and cancer therapy. Further, data is limited on the immune landscape of cancer patients; whether components of cellular and humoral immunity are compromised, the impact of immune-modulating therapies such as B cell depleting therapy, and how these factors influence mortality in the setting of COVID-19 is also not known. To address these questions, we studied three cohorts of cancer patients with acute COVID-19 across two hospital systems to understand the immunologic determinants of COVID-19 mortality in cancer.

## Hematologic cancer is an independent risk factor of COVID-19 mortality

To understand the clinical impact of COVID-19 on cancer patients, we first conducted a prospective multi-center observational cohort study of cancer patients hospitalized with COVID-19 (COVID-19 Outcomes in Patients with Cancer, COPE). Between April 28 and September 15 2020, 114 patients with history of hematologic or solid tumor malignancy, and laboratory-confirmed SARS-CoV-2 infection or presumed COVID-19 diagnosis, were enrolled across 4 hospitals in the University of Pennsylvania Health System. 14 patients were excluded from the analyses due to either low suspicion for COVID-19 infection, or benign tumor diagnosis. The median age of this cohort was 68 years, 52% were male, 54% Black, and 57% were current or former smokers (Table 1), reflecting the demographics of severe COVID-19^38, 39^. In terms of cancer-specific factors, 78% of patients had solid cancers, with prostate and breast cancers most prevalent; 46% had active cancer, defined as diagnosis or treatment within 6 months; and 49% had a recorded ECOG performance status of 2 or higher (Table 1). During follow up, 48% of subjects required ICU level care, and 38% of patients died within 30 days of admission (Table 2), consistent with previously reported rates for severe COVID-19 in this population^30, 34, 37^.

**Fig. 1 |:**
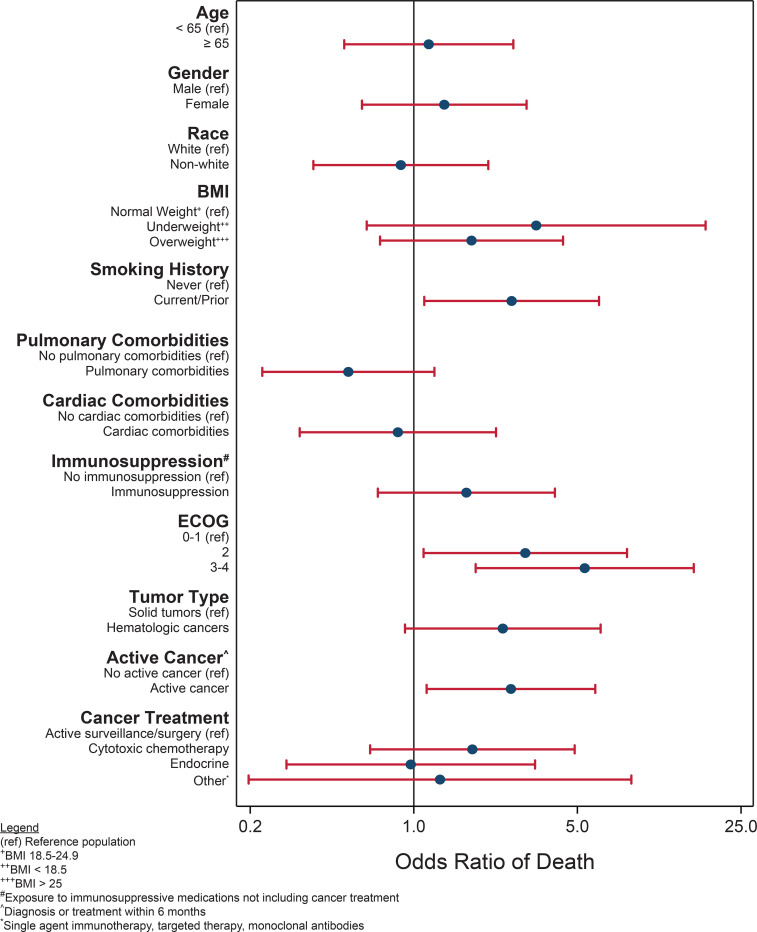
Univariate analysis of potential risk factors in COVID-19 mortality.

To understand key determinants of COVID-19 disease severity, we performed univariate analysis to identify factors associated with all-cause mortality within 30 days of discharge. We included relevant covariates, including patient factors such as age, race, gender, and smoking history (ever versus never)^2, 38–40^; cancer-specific factors including ECOG performance status^35^, status of cancer (e.g., active versus remission)^36, 36^; cancer type (e.g., heme versus solid cancer)^29, 34, 36, 41, 42^; and cancer treatment^26, 37^. Increased mortality was significantly associated with prior or current smoking (p = 0.028), poor ECOG performance (ECOG 3–4, p=0.001), and active cancer status (p=0.024) (Fig. 1). In addition, patients with hematologic cancers (mostly lymphoma and leukemia), appeared to have an increased risk of mortality relative to solid cancers (54% versus 33% respectively, p=0.075) (Table 3). This is consistent with recent data showing increased disease severity and mortality in patients with hematologic malignancies^23, 29, 34–36, 41^. Notably, cancer treatment, including cytotoxic chemotherapy, was not significantly associated with COVID-19 mortality, also consistent with published literature in patients with cancer^29, 30, 34, 36, 41^.

**Extended Data Fig. 1 |:**
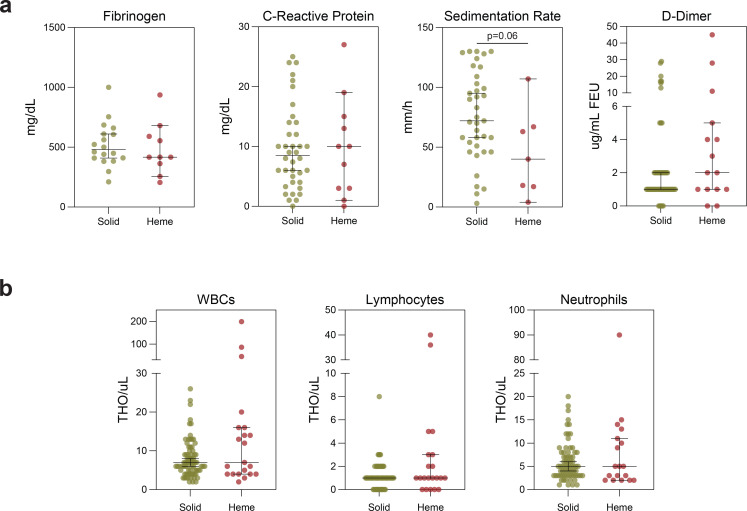
Inflammatory markers and blood cell counts in cancer patients with COVID-19. Clinical laboratory values for (**a**) inflammatory markers and (**b**) cell counts in solid (n=62) and hematologic (n=21) cancer patients. (All) Significance determined by Mann Whitney test: *p<0.05, **p<0.01, ***p<0.001, and ****p<0.0001. Median and 95% CI shown.

**Fig. 2 |:**
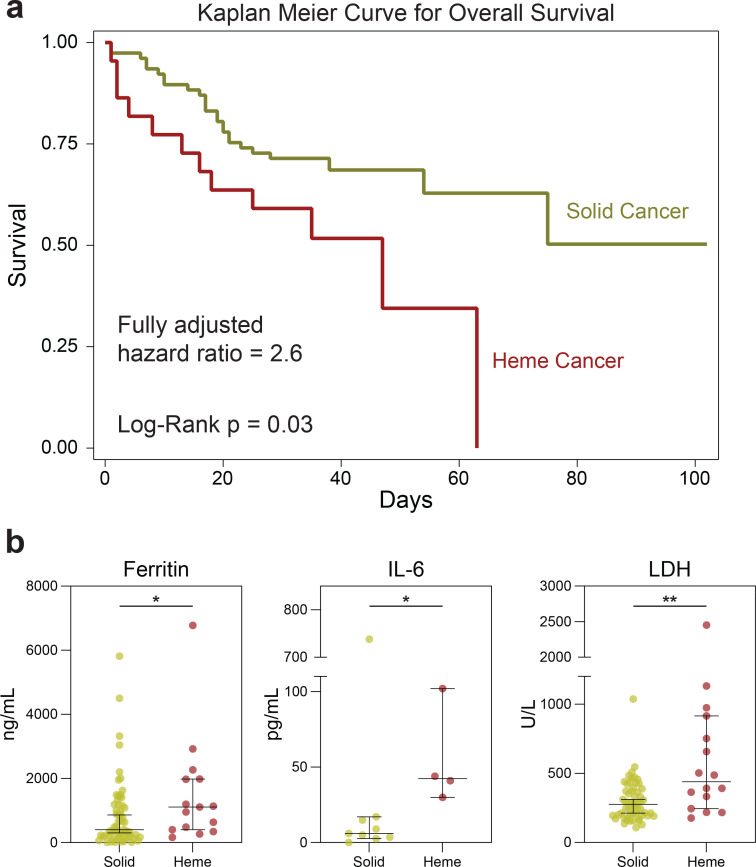
Hematologic cancer is an independent risk factor for COVID-19 related mortality. (**a**) Kaplan Meier curve for COVID-19 survival of patients with solid (n=77) and hematologic (n=22) cancer. Cox regression-computed hazard ratio for mortality in hematologic vs solid cancer, adjusted for age, gender, smoking status, active cancer status, and ECOG performance status. (**b**) Ferritin, IL-6, and LDH in solid (n=62) and hematologic (n=15) cancer hospitalized for COVID-19. (All) Significance determined by Mann Whitney test: *p<0.05, **p<0.01, ***p<0.001, and ****p<0.0001. Median and 95% CI shown.

To determine whether the increased mortality observed in patients with hematologic malignancy was independent of potential confounding effects from smoking history, poor ECOG performance, and active cancer, which were not corrected for in the prior studies, we performed multivariable logistic regression. Patients with hematologic cancers tended to be younger, male, less likely to have coexisting comorbidities, and more likely to have received recent cytotoxic chemotherapy (Supplemental Table 1). In this fully adjusted analysis, hematologic malignancy was strongly associated with mortality, in comparison to solid cancer (OR 3.3, 95% CI 1.01–10.8, p=0.048) (Table 3). Similar results were observed in time-to-event analyses using Kaplan Meyer methods (Fig. 2a, median overall survival (mOS) not reached for patients with solid cancers vs 47 days for patients with heme cancers, p-value=0.030) and Cox regression models (Table 3, HR 2.56, 95% CI 1.19–5.54, p=0.017). Moreover, patients with hematologic cancers had higher levels of many inflammatory markers on admission laboratory testing, including ferritin, IL-6, and LDH (Fig. 2b). There were no significant differences in CRP, fibrinogen, D-dimer, lymphocyte counts, and neutrophil counts, while ESR was higher in patients with solid cancer (Extended Data Fig. 1 a,b). Thus, hematologic malignancy was an independent risk factor of death, with signs of a dysregulated inflammatory response.

## Hematologic cancer patients have an impaired SARS-CoV2-specific antibody response.

**Fig. 3 |:**
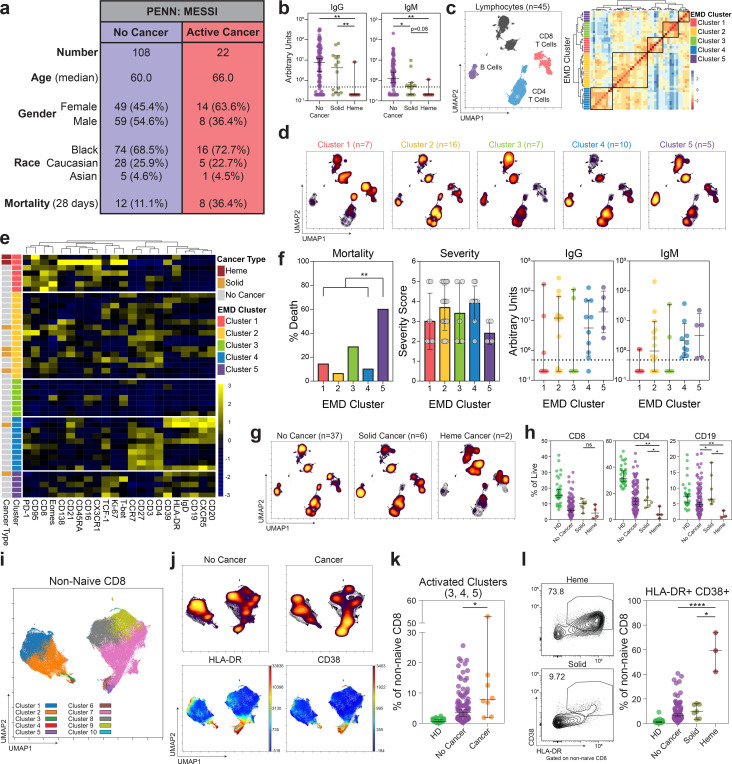
High dimensional analyses reveal immune phenotypes associated with mortality and distinct phenotypes between solid and hematologic cancers. (**a**) Demographic and mortality data for MESSI cohort at Penn. (**b**) Relative levels of SARS-CoV-2 IgG and IgM of solid (n=14) and hematologic (n=7) cancer patients and non-cancer patients (n=108). (**c**) (Left) Global UMAP projection of lymphocyte populations for all 45 patients pooled. (Right) Hierarchical clustering of Earth Mover’s Distance (EMD) using Pearson correlation, calculated pairwise for lymphocyte populations. (**d**) UMAP projection of concatenated lymphocyte populations for each EMD cluster. (Yellow: High Density; Black; Low Density) (**e**) Heatmap showing expression patterns of various markers, stratified by EMD cluster. Heat scale calculated as column z-score of MFI. (**f**) Mortality, disease severity, and SARS-CoV-2 antibody data, stratified by EMD cluster (Cluster 5 n=5; Cluster 1,2,3,4 n=40). Mortality significance determined by Pearson Chi Square test. Severity assessed with NIH ordinal scale for COVID-19 clinical severity (1: Death; 8: Normal Activity)^15^. (**g**) UMAP projections of concatenated lymphocyte populations for solid cancer, hematologic cancer, and non-cancer patients. (**h**) CD8 and CD4 T cell and B cell frequencies in healthy donors (HD) (n=33), non-cancer (n=108), solid cancer (n=7), and heme cancer (n=4). (**i**) UMAP projection of non-naive CD8 T cell clusters identified by FlowSOM. (**j**) (Top) UMAP projections of non-naïve CD8 T cells for non-cancer and cancer patients. (Bottom) UMAP projections indicating HLA-DR and CD38 protein expression on non-naive CD8 T cells for all patients pooled. (**k**) Frequency of activated FlowSOM clusters in HD (n=30), non-cancer (n=110), and cancer patients (n=8). (**l**) Representative flow plots and frequency of HLA-DR and CD38 co-expression in HD (n=30), non-cancer (n=110), solid cancer (n=7), and hematologic cancer (n=3) patients. (All) Significance determined by Mann Whitney test: *p<0.05, **p<0.01, ***p<0.001, and ****p<0.0001. Median and 95% CI shown.

To understand the immune landscape in cancer patients, as compared to patients without cancer, we leveraged an observational study of hospitalized COVID-19 patients at the University of Pennsylvania Health System where blood was collected (MESSI-COVID)^15^. This analysis included 130 subjects with flow cytometric and/or serologic analysis. In particular, we focused on 22 subjects with active cancer (Supplemental Tables 2, 3), including patients undergoing cancer-directed therapies such as chemotherapy, immunotherapy, or B cell directed therapies (Supplemental Table 4). Age, gender, and race were similarly distributed in COVID-19 patients with active cancer and those without, and both groups had a similar timeframe of symptom onset and disease severity (Fig. 3a, Supplemental Table 2). However, cancer patients had a higher all-cause mortality (36.4% versus 11.1%, Fig. 3a), consistent with our COPE clinical cohort, and what has been reported in other cohorts of COVID-19 patients^23, 26, 29, 30^.

**Extended Data Fig. 2 |:**
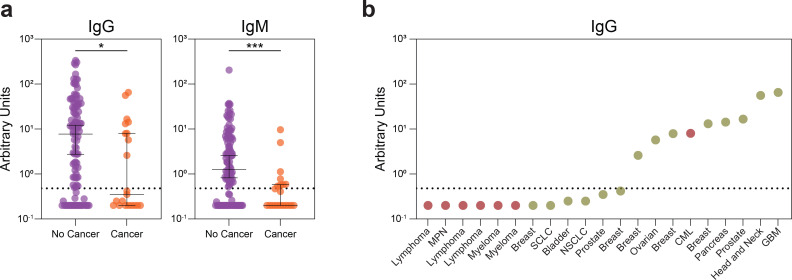
SARS-CoV-2 antibody levels. (**a**) Relative levels of SARS-CoV-2 IgG and IgM in non-cancer (n=108) and cancer (n=21) patients. (**b**) Relative IgG levels in cancer patients. Each dot represents a cancer patient (Heme: Red; Solid: Yellow). (All) Significance determined by Mann Whitney test: *p<0.05, **p<0.01, ***p<0.001, and ****p<0.0001. Median and 95% CI shown.

As humoral immunity is critical for protective immunity against SARS-CoV-2, we hypothesized that a defect in SARS-CoV-2-specific antibodies may be associated with the increase in mortality seen in patients with active cancer. We assessed the levels of IgM and IgG antibodies that recognized the SARS-CoV-2 receptor binding domain (RBD), using an enzyme-linked immunosorbent assay (ELISA) based approach^43, 44^. Cancer patients had significantly decreased SARS-CoV-2-specific IgG and IgM responses compared to non-cancer patients (Extended Data Fig. 2a). These differences were not due to the timing of SARS-CoV-2 infection as time from symptom onset was similar (Supplemental Table 2). As hematologic malignancies directly involve the lymphoid and myeloid immune compartments, we suspected that hematologic cancers may have an impaired humoral immunity against SARS-CoV-2. Indeed, the vast majority of hematologic cancer patients (6/7) had IgM and IgG levels below the cutoff of positivity of 0.48 arbitrary units (Fig. 3b, Extended Data Fig. 2). In contrast, those with solid cancers had IgG and IgM antibody responses that were more comparable to patients without cancer (Fig. 3b).

## A T cell-depleted immune phenotype is associated with COVID-19 mortality.

**Extended Data Fig. 3 |:**
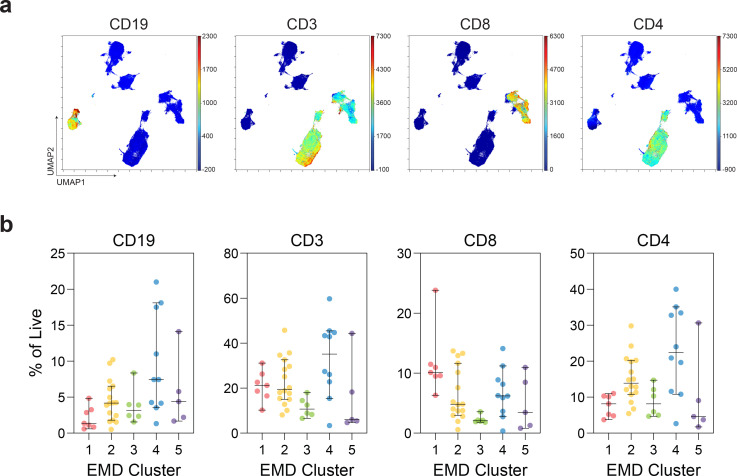
Dimensionality reduction and EMD clustering of MESSI cohort. (**a**) UMAP projections of lymphocytes with indicated protein expression. (**b**) Frequencies of CD19+, CD3+, CD3+CD8+, and CD3+CD4+ cells of patients in each EMD cluster (Cluster 1 n=7; Cluster 2 n=16; Cluster 3 n=6; Cluster 4 n=10; Cluster 5 n=5). (All) Median and 95% CI shown.

Protective antibody responses require effective T cell and B cell responses. We therefore examined whether cancer patients had an altered cellular response to SARS-CoV-2. We first performed exploratory high-dimensional analysis on the lymphocyte compartment of 45 patients with COVID-19 including 37 non-cancer, 6 solid cancer, and 2 hematologic cancer patients. UMAP (Uniform Manifold Approximation and Projection) representation of 27-parameter flow cytometry data highlighted discrete islands of CD4 and CD8 T cells, and CD19+ B cells (Extended Data Fig. 3a and Fig. 3c). To understand whether there were major global differences in lymphocytes between solid, hematologic, and non-cancer patients, we used the Earth Mover’s Distance (EMD) metric^45^ to calculate the distance between the UMAP projections for every pair of patients. Clustering on EMD values identified 5 clusters of patients with similar lymphocyte profiles (Fig. 3c). Differences between these clusters of patients were driven by both the distribution (Fig. 3d) and phenotype (Extended Data Fig. 3b and Fig. 3e) of CD4, CD8, and B cells. EMD cluster 1 was defined by depleted CD4 and B cells, increased CD8 T cells, and increased activation and effector markers, including PD-1, CX3CR1, Ki67, and HLA-DR (Extended Data Fig. 3b and Fig. 3 d,e). EMD cluster 3 had decreased T cell and B cells, with an inactivated immune profile, and EMD Cluster 5 was depleted of both CD4 and CD8 T cells, but had preserved B cells. In contrast, EMD cluster 4 was defined by robust CCR7+CD27+ memory CD4 T cell responses and heterogenous B cell responses; EMD cluster 2 had the most balanced responses, with CD4, CD8, and B cells represented (Fig. 3d,e and Extended Data Fig. 3b). We then correlated these 5 patterns of immune responses with clinical and serological variables. EMD cluster 5 patients with depleted T cells had the highest mortality and disease severity, despite generating SARS-CoV-2-specific IgM and IgG antibodies (Fig. 3f, Extended Data Fig. 5d). In contrast, EMD clusters 2 and 4, with robust CD4 and/or CD8 T cell responses, had the lowest mortality and a low disease severity (Fig. 3f, Extended Data Fig. 5d). These findings suggest a key role for T-cell immunity in facilitating viral clearance, even in the presence of intact humoral immunity.

## Distinct immune landscape in hematologic cancer compared to solid cancer or no cancer.

**Extended Data Fig. 4 |:**
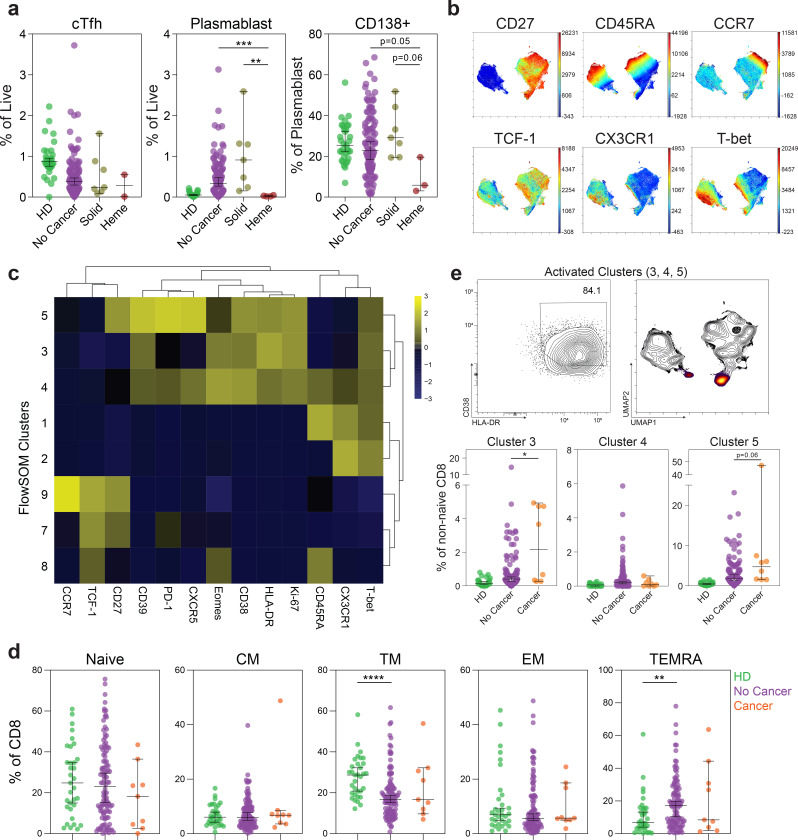
Cellular phenotyping of COVID-19 patients with cancer. (**a**) Frequencies of circulating T follicular helper cells (cTfh), plasmablasts, and CD138 expression on plasmablasts (HD n=33; non-cancer n=108; solid cancer n=7; heme cancer n=3). (**b**) UMAP projection of non-naïve CD8 T cells with indicated protein expression. (**c**) Heatmap showing expression patterns of various markers, stratified by FlowSOM clusters. Heat scale calculated as column z-score of MFI. (**d**) Frequencies of CD8 subsets: naive (CD45RA+CD27+CCR7+), central memory (CD45RA−CD27+CCR7+), transition memory (CD45RA−CD27+CCR7−), effector memory (CD45RA−CD27−CCR7−), and TEMRA (CD45RA+CD27−CCR7−) (HD n=33; non-cancer n=108; cancer n=9). (**e**) (Top) HLA-DR and CD38 coexpression in concatenated activated clusters (3, 4, and 5) and associated UMAP localization. (Bottom) Frequency of activated clusters (3, 4, and 5) in each patient (HD n=30; non-cancer n=110; solid-cancer n=8). (All) Significance determined by Mann Whitney test: *p<0.05, **p<0.01, ***p<0.001, and ****p<0.0001. Median and 95% CI shown.

To further understand the immune response of patients with cancer and COVID-19, we explored the role of cancer subtype (solid tumor versus hematologic) on immune phenotype. Four out of the 6 solid cancer patients were in EMD cluster 2, with a balanced immune phenotype (Fig. 3e). In contrast, both hematologic cancer patients were in EMD cluster 1, which had marked depletion of CD4 and B cells. Indeed, UMAP projections showed that while solid cancer patients had an immune landscape similar to non-cancer patients, the two hematologic cancer patients demonstrated loss of islands associated with CD4 and B cells (Fig. 3g). We then extended this analysis by measuring the frequency and phenotype of key lymphocyte populations in the entire MESSI-COVID cohort and healthy donor controls. COVID-19 patients with hematologic cancers had a significantly lower frequency of CD4 and B cells compared to solid cancer patients, non-cancer patients, and healthy donors without COVID-19 (Fig. 3h). As T follicular helper cells (Tfh) and plasmablasts are critical in the generation of effective antibody responses, we assessed circulating Tfh and plasmablast responses. Although limited by sample size, patients with hematologic cancers had low circulating Tfh (PD1+ CXCR5+) and plasmablast responses (CD19+CD27^hi^CD38^hi^), and decreased CD138 expression (Extended Data Fig. 4a). Thus, patients with hematologic malignancy appear to have quantitative defects in CD4 and B cells that may be required for effective SARS-CoV-2-specific antibody responses.

Patients with hematologic cancers had a preserved frequency of CD8 T cells. Therefore, we wanted to determine whether there were qualitative changes within the CD8 T cell compartment. We performed FlowSOM clustering analysis on non-naïve CD8 T cells from 118 COVID-19 patients and 30 healthy donors and visualized the clusters using UMAP. UMAP clearly separated CX3CR1 and Tbet expressing effector cells from memory CD8 T cells expressing CD27 and TCF-1 (Extended Data Fig. 4b and Fig. 3i). The effector island was composed of CD45RA^lo^CD27^lo^ effector memory cells (clusters 2 and 3) and CD45RA+ TEMRA cells (cluster 1). The memory island was composed of CCR7^lo^ transitional memory (cluster 5), and effector memory cells (clusters 7 and 8), and CCR7^hi^ central memory cells (cluster 9). Activated cells, characterized by high HLA-DR, CD38, and Ki67 expression, were identified in clusters 3, 4, and 5 (Extended Data Fig. 4c). Stem cell memory cells (cluster 10) and exhausted phenotype CD8 T cells (cluster 6) were present, but at low frequencies of below 0.5% (Data not shown).

We then compared the landscape of CD8 T cells in patients with and without cancer. CD8 T cell subsets including central memory, effector memory, transitional memory and EMRA, were similar between patients with and without cancer (Extended Data Fig. 4d). However, UMAP representation of non-naïve CD8 data demonstrated preferential enrichment of cells expressing HLA-DR and CD38 in cancer patients compared to non-cancer patients (Fig. 3j). Indeed, cancer patients had higher frequencies of activated HLA-DR, CD38, and Ki67-expressing FlowSOM clusters (clusters 3, 4, and 5) compared to non-cancer patients and healthy donors (Extended Data Fig. 4e and Fig. 3k). When stratified by cancer type, the increased HLA-DR and CD38 expression was restricted to the patients with hematologic cancers; patients with solid cancers and those without cancer had comparable levels of activation (Fig. 3l). Altogether, solid cancer patients with COVID-19 had an immune landscape similar to non-cancer COVID-19 patients. In contrast, patients with hematologic malignancies had defects in CD4, B cells, and humoral immunity but preserved and highly activated CD8 T cells, suggesting that CD8 T cells might at least partially compensate for blunted humoral immune responses in patients with hematologic malignancies.

## CD8 T cell adequacy increases survival in the setting of impaired B cell and humoral immunity in hematologic cancer.

**Extended Data Fig. 5 |:**
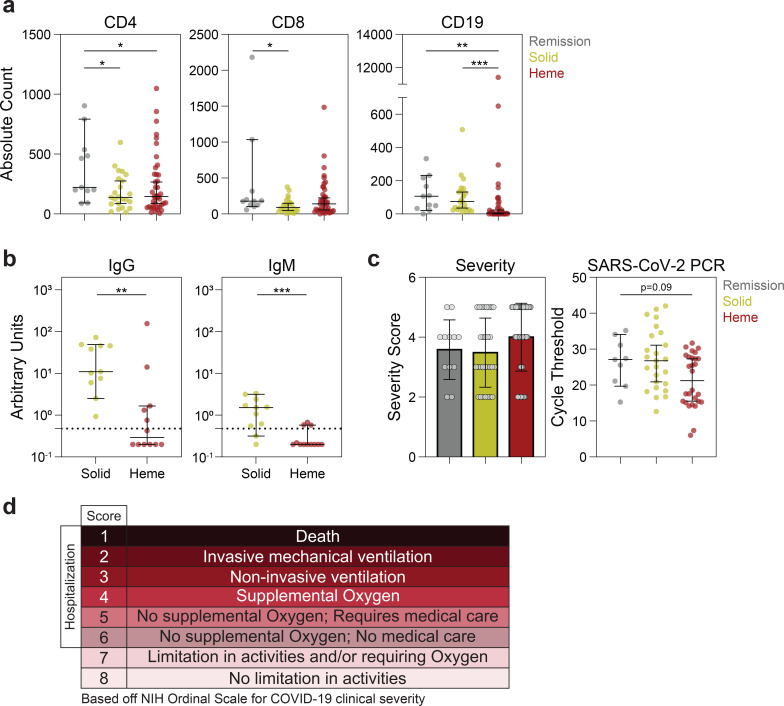
Cellular, serologic, and clinical features in solid and hematologic cancer patients with COVID-19. (**a**) Absolute counts of CD4, CD8, and CD19 expression in remission (n=11), solid cancer (n=23), and hematologic cancer (n=41) patients. (**b**) Relative levels of SARS-CoV-2 IgG and IgM in solid (n=11) and hematologic cancer (n=14) patients. (**c**) Severity (NIH ordinal scale for COVID-19 clinical severity) and RT-PCR cycle threshold (remission n=9; solid n=25; heme n=28) (Lower Ct: Higher viral load). (d) NIH ordinal scale for COVID-19 clinical severity. (All) Significance determined by Mann Whitney test: *p<0.05, **p<0.01, ***p<0.001, and ****p<0.0001. Median and 95% CI shown.

**Fig. 4 |:**
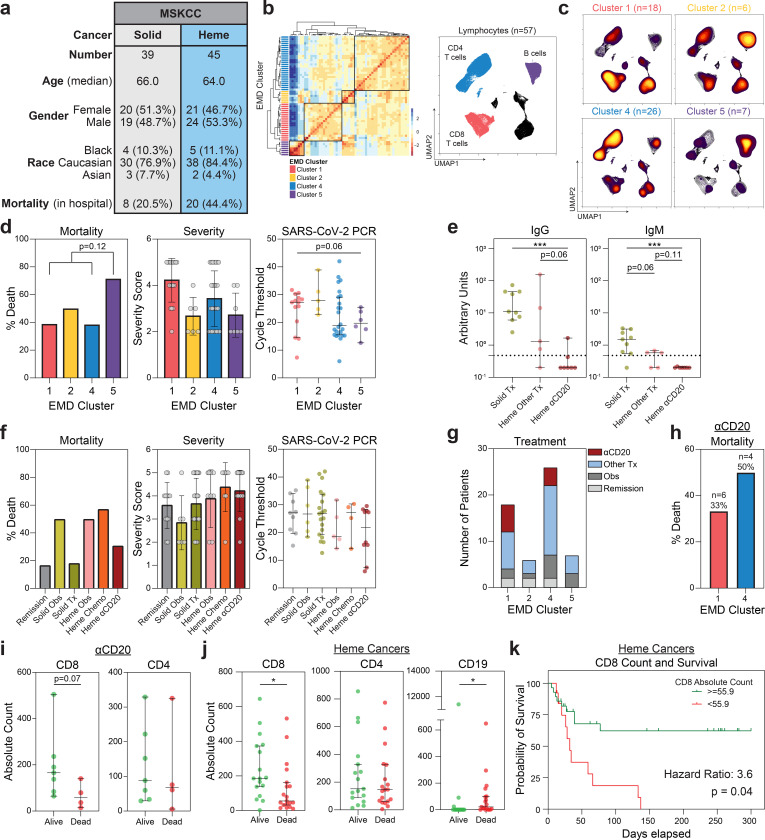
CD8 T cell counts associated with survival in hematologic cancer patients with COVID-19. (**a**) Demographic and mortality data of MSKCC cohort. (**b**) (Left) Hierarchical clustering of Earth Mover’s Distance (EMD) using Pearson correlation, calculated pairwise for lymphocyte populations. (Right) Global UMAP projection of lymphocyte populations pooled. (**c**) UMAP projection of concatenated lymphocyte populations for each EMD cluster. (Yellow: High Density; Black: Low Density) (**d**) Mortality (Cluster 5 n=7; Cluster 1,2,4 n=50), severity, and RT-PCR cycle threshold (Cluster 1 n=14; Cluster 2 n=5; Cluster 4 n=24; Cluster 5 n=6) (Lower Ct: Higher viral load) stratified by EMD cluster. Mortality significance determined by Pearson Chi Square test. (**e**) Relative levels of SARS-CoV-2 IgG and IgM of patients with recent cancer treatments (solid tx n=9; heme αCD20 n=7; heme other tx n=5). (**f**) Mortality, severity, and RT-PCR cycle threshold stratified by cancer treatment (remission n=9; solid obs n=6; solid tx n=19; heme obs n=5; heme chemo n=4; heme αCD20 n=10). Severity assessed with NIH ordinal scale for COVID-19 clinical severity. (**g**) Recent cancer treatment of patients in each EMD cluster. (**h**) Mortality of patients treated with B cell depleting therapy in EMD cluster 1 (red) and EMD cluster 4 (blue). (**i**) Absolute CD8 and CD4 T cell counts in patients treated with B cell depleting therapy (alive n=7; dead n=4). (**j**) Absolute CD8 and CD4 T cell counts and B cell counts in hematologic cancer patients (alive n=17; dead n=18). (**k**) Kaplan-Meier curve for survival in hematologic cancer patients stratified by CD8 T cell counts (threshold = 55.9; log-rank hazard ratio) (>=55.9 n=28; <55.9 n=13). CD8 count threshold determined by Classification and Regression Tree (CART) analysis. (All) Significance determined by Mann Whitney test: *p<0.05, **p<0.01, ***p<0.001, and ****p<0.0001. Median and 95% CI shown.

Patients with hematologic cancer had significantly impaired humoral immunity and a mortality rate of 55% (Table 2). We hypothesized that CD8 T cells partially compensated for defective humoral immunity and influenced survival in acute COVID-19. We tested this hypothesis in a cohort of cancer patients hospitalized with COVID-19 at the Memorial Sloan Kettering Cancer Center (MSKCC), which included a larger number of hematologic malignancies patients, including those treated with B cell depleting therapy. This cohort included 39 solid cancer patients and 45 hematologic cancer patients. The median age was 65 years, and in contrast to the MESSI cohort at Penn, 81% of the cohort was white (Fig. 4a, Supplementary Table 5,6). A significant portion of patients were treated with remdesevir and convalescent plasma – 21.4%, and 46.4%, respectively (Supplementary Table 5). Consistent with the Penn COPE and MESSI cohorts, patients with hematologic cancers did poorly, with a mortality rate of 44.4% (Fig. 4a, Supplementary Table 5). Clinical grade 12-parameter flow cytometry and serologic testing for SARS-CoV-2-specific antibodies were performed. In the MSKCC cohort, both CD4 and CD8 T cells were significantly decreased in patients with active solid and hematologic cancers, compared with patients in clinical remission (Extended Data Fig. 5a). Moreover, despite the fact that a substantial number of patients with hematologic cancers from the MSKCC cohort received convalescent plasma, they had a significant defect in SARS-CoV-2-specific IgG and IgM responses as compared to solid cancers (Extended Data Fig. 5b). This was independent of disease severity and viral load, as assessed by RT-PCR cycle threshold. (Extended Data Fig. 5c,d).

**Extended Data Fig. 6 |:**
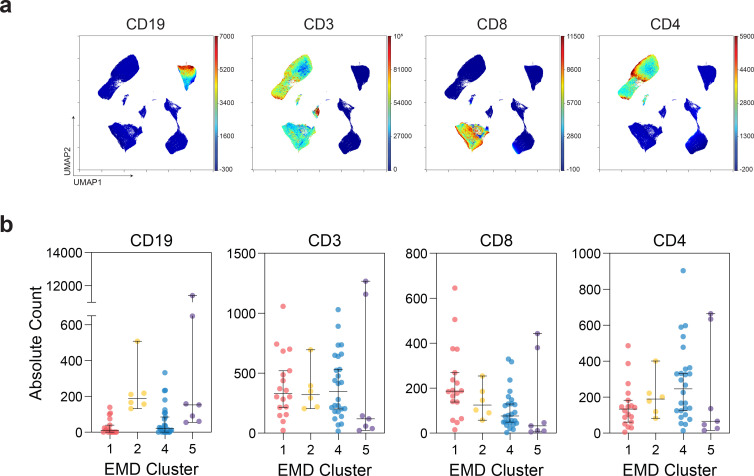
Dimensionality reduction and EMD clustering of MSKCC cohort. (**a**) UMAP projections of lymphocytes with indicated protein expression. (**b**) Absolute counts of CD19+, CD3+, CD3+CD8+, and CD3+CD4+ cells of patients in each EMD cluster (Cluster 1 n=18; Cluster 2 n=6; Cluster 3 n=26; Cluster 4 n=7). (All) Median and 95% CI shown.

We performed high dimensional analyses on flow cytometry data that included information on CD4 T cells, CD8 T cells, and B cells. EMD and clustering of 20 solid cancer, 31 hematologic cancer, and 6 remission patients identified 4 immune phenotypes (Extended Data Fig. 6a,b and Fig. 4b,c) that corresponded to the immune phenotypes 1,2,4, and 5 identified in the Penn-MESSI cohort (Fig. 3c,d). The Penn phenotype 3, the only cluster that did not have cancer patients, was not identified in the MSKCC cancer cohort. Consistent with the Penn data, MSKCC EMD cluster 5, with depleted of CD4 and CD8 T cells and preserved B cells, had the highest mortality of 71%, and was associated with a high disease severity and viral load (Fig 4d).

**Extended Data Fig. 7 |:**
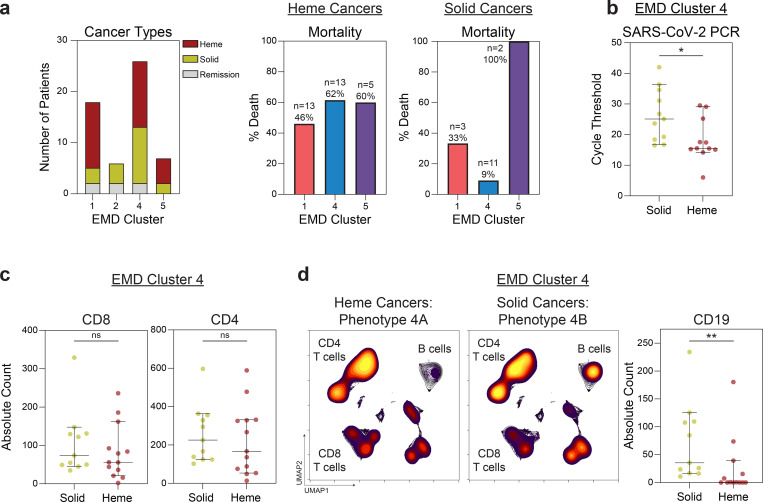
EMD Cluster 4 drives differences in mortality between hematologic and solid cancer patients. (**a**) (Left) Number of patients with hematologic, solid, and remission cancer statuses within each EMD cluster. (Right) Mortality of patients within each EMD cluster for hematologic and solid cancers. (**b**) RT-PCR cycle threshold of solid and heme cancer patients in EMD cluster 4 (solid n=11; heme n=11). (**c**) Absolute CD8 and CD4 T cell counts for subjects in EMD cluster 4 stratified by solid (n=11) and heme (n=13) cancer. (**d**) Global UMAP projections of lymphocytes for subjects in EMD cluster 4: (Left) Hematologic cancer; (Middle) Solid cancer. (Right) Absolute B cell counts for subjects in EMD cluster 4 stratified by solid (n=11) and heme (n=13) cancer. (All) Significance determined by Mann Whitney test: *p<0.05, **p<0.01, ***p<0.001, and ****p<0.0001. Median and 95% CI shown.

Intriguingly, the clinical outcomes of patients with immune phenotype 4 was the greatest contributor to the overall mortality difference between patients with solid and liquid cancers; hematologic cancer patients with phenotype 4 had a mortality of 61% versus 9% in patients with solid cancers (Extended Data Fig. 7a), with a corresponding higher viral load as assessed by RT-PCR threshold cycle (Extended Data Fig. 7b). Immune phenotype 4 was characterized by robust CD4 responses and decreased, but still intact, CD8 responses (Extended Data Fig 6b). Within immune phenotype 4, patients with solid and hematologic cancers had similar CD4 and CD8 T cell counts (Extended Data Figure 7c). However, patients with hematologic cancers had near-complete abrogation of B cells (phenotype 4A), that corresponded with a mortality rate of 61% (Extended Fig 7a and d). In contrast, patients with solid cancers had intact B cells counts (phenotype 4B, Extended Data Fig 7a and d), with a mortality of 9%. Thus, in a setting with similar CD4 and CD8 T cell numbers, B cell depletion was associated with higher mortality; B cells, therefore, likely play an important role in acute COVID-19.

**Extended Data Fig. 8 |:**
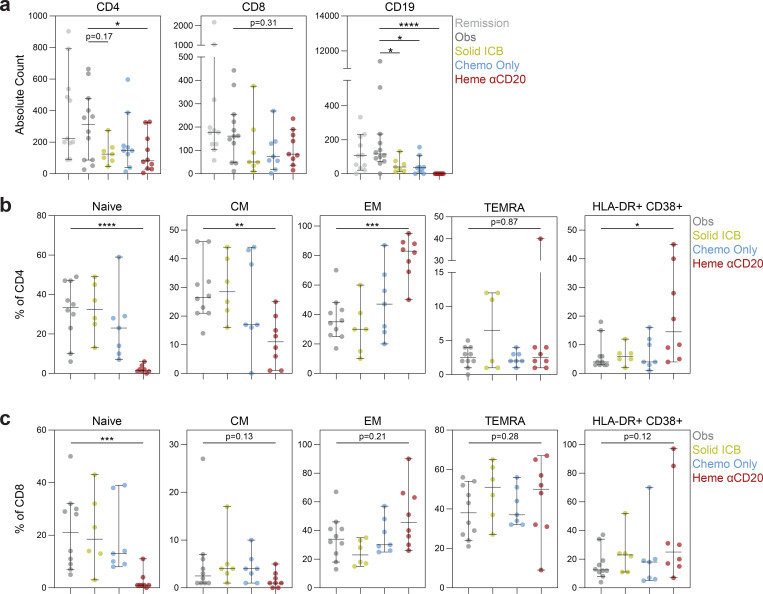
Effect of cancer treatment on T cell differentiation in cancer patients with COVID-19. (**a**) Absolute counts of CD4, CD8, and CD19 expressing cells. Frequencies of (**b**) CD4 and (**c**) CD8 T cell subsets in cancer patients treated with immune checkpoint blockade therapies, chemotherapies, and B cell depleting therapies. Naive (CD45RA+CCR7+), CM (CD45RA−CCR7+), EM (CD45RA−CCR7−), TEMRA (CD45RA+CCR7−). (All) Remission n=11, obs n=12, chemo only n=9, solid ICB n=7, and heme αCD20 n=10. Significance determined by Mann Whitney test: *p<0.05, **p<0.01, ***p<0.001, and ****p<0.0001. Median and 95% CI shown.

Anti-CD20 therapy (αCD20) with rituximab or obinutuzumab-containing regimens depleted B cells with near-complete abrogation of SARS-CoV-2-specific IgG and IgM responses (Fig. 4e). Notably, hematologic cancer patients on chemotherapy and solid cancer patients on immune checkpoint blockade also had significant depletion of B cells (Extended Data Fig. 8a). αCD20 therapy was not associated with quantitative changes in CD4 and CD8 T cells. However, patients treated with anti-CD20 therapy displayed dramatic reduction in CD4 and CD8 naïve and memory T cells, instead skewing towards effector differentiation and an activated HLA-DR+CD38+ phenotype (Extended Data Fig. 8b,c). Importantly, despite the loss of B cells and humoral immunity, αCD20 therapy was not associated with increased mortality, disease severity, or viral load when compared to chemotherapy or observation (Fig. 4f).

**Extended Data Fig. 9 |:**
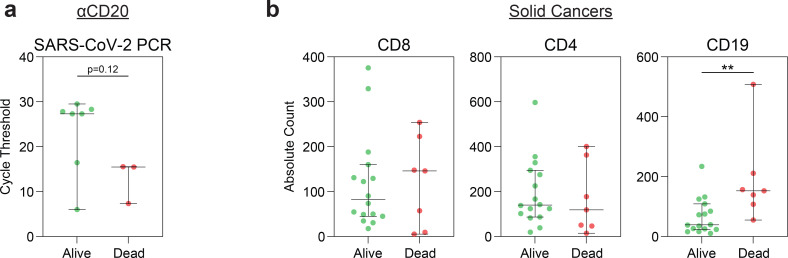
Association of mortality with cell counts and viral load. (**a**) RT-PCR cycle threshold of patients treated with αCD20 therapy (alive n=7; dead n=3). (**b**) Absolute counts of CD8+, CD4+, and CD19+ cells in solid cancer patients (alive n=16; dead n=7). (All) Significance determined by Mann Whitney test: *p<0.05, **p<0.01, ***p<0.001, and ****p<0.0001. Median and 95% CI shown.

We sought to understand why αCD20 therapy was not associated with greater mortality in these patients. Patients treated with αCD20 therapy were restricted to immune phenotypes 1 and 4, characterized by depleted B cells (Fig 4g). However, phenotype 1, characterized by preserved CD8 T cells, was associated with a lower mortality (Fig 4h). Indeed, αCD20 treated patients who survived their COVID-19 hospitalization had higher CD8 T cell counts (Fig 4i), and lower viral load (Extended Data Fig. 9a). We extended these analyses to other patients with hematologic cancers, including those on chemotherapy who also had quantitative (Extended Data Fig. 8a), and possibly qualitative B cell defects. Hematologic cancer patients who survived had higher CD8 T cell count (Fig. 4j), which was not seen in solid cancer patients (Extended Data Fig. 9b). Conversely, CD4 T cell counts were not associated with mortality, and higher B cell counts were associated with increased mortality (Extended Data Fig. 9b, Fig 4j). Thus, patients with hematologic cancers, in the setting of defective humoral immunity, were more highly dependent on adequate CD8 T cell counts than patients with solid cancers. Finally, Classification and Regression Tree Analysis (CART) identified a CD8 T cell level that was predictive of survival after COVID-19 in patients with hematologic cancers (Fig. 4k). Taken together, these findings suggest that CD8 T cells are critical for anti-viral immunity in hematologic malignancy patients and may at least partially mitigate the negative impact of B-cell depletion on COVID outcomes.

## Discussion

A notable feature of the COVID-19 pandemic has been the dramatic heterogeneity in clinical presentations and outcomes, yet mechanistic explanations for the wide variance in disease severity have remained elusive. Early on, acute phase reactants and systemic cytokines were implicated in patient outcomes^46^ and hospital stay and mortality were decreased by dexamethasone^47^, suggesting that an excessive host immune response might contribute to COVID-19 mortality. However, there were also indications that inadequate host immunity might contribute to adverse COVID-19 outcomes, including the association of lymphopenia with mortality as well as the potentially inferior outcomes of patients on chronic immunosuppression, such as patients with autoimmune diseases or organ transplant recipients^48–51^. Recent studies defined immune signatures associated with severe COVID-19, including activated CD4, CD8 T cells, plasmablasts, and robust antibody responses^15, 16, 20, 52^. Nevertheless, the individual roles of these cell types in acute COVID-19 remained unclear. We speculated that investigating both the clinical outcomes and immunologic profile of cancer patients might shed valuable insight into how arms of the immune system contribute to viral control and mortality during COVID-19. Immune investigation in hematologic malignancies is especially relevant because the disease directly impacts the lymphoid and myeloid immune cells, and is commonly treated with myelosuppressive and B cell-depleting therapies including CD20 targeting antibodies.

Our investigation reveals several novel findings. First, we establish in a prospective clinical cohort that hematologic malignancy is an independent predictor of COVID-19 mortality after adjusting for ECOG performance and disease status. We observed a higher mortality rate in patients with hematologic (53%) versus solid cancers (34%), which were substantially higher than in the general population (2.7%)^28^. The high mortality rates for hematologic cancer in this study were consistent with a recent meta-analysis of 2,361 hospitalized patients with hematologic cancer (40%)^53^. This finding highlights the importance of transmission mitigation efforts for this vulnerable population^54^. Furthermore, we demonstrate that excess mortality observed with hematologic cancers persisted (HR 2.5) after adjustment for independent predictors of cancer mortality, including age, smoking history, poor performance status, and active or advanced disease. Adjustment for these factors was necessary to determine that the increased mortality difference seen in hematologic cancer was in fact, driven by cancer subtype, rather than differences in patient characteristics. These data can better inform hospitalized patients with hematologic cancers of their expected outcomes, irrespective of performance status or active cancer status, thereby improving decision-making between best supportive care or aggressive interventions. The disease-specific increased risk of COVID-19 associated mortality in hematologic cancer patients may also influence the prioritization and distribution of vaccinations to this very high-risk population.

Second, using high dimensional analyses, we define immune phenotypes associated with mortality during COVID-19. In particular, we identify the immune phenotype that drives the mortality difference between solid and liquid malignancy. A balanced immunity that included CD4, CD8, and B cells responses (phenotypes 2 and 4b) was associated with low mortality. In contrast, an immune signature with robust B cell and humoral responses, but absent T cell responses (phenotype 5), was associated with the highest mortality (>60%). A high mortality for patients with immune phenotype 5 was consistent in both the Penn and MSKCC cohorts, and in patients with solid cancer, hematologic cancer, and infected patients without cancer. Thus, humoral immunity alone is often not sufficient in acute COVID-19. In fact, greater B cell responses was associated with higher mortality in both solid and liquid cancer. B cell responses may be a marker of disease severity, as seen with plasmablasts^15, 20^ and neutrophils^20, 55, 56^ in severe COVID-19. Alternatively, some components of the B cell and humoral responses may be aberrant and pathogenic, as may be the case with autoantibodies targeting type I interferons in severe COVID-19^57^.

Consistent with recent data^58^, patients with solid cancers had a similar cellular immune landscape and SARS-CoV-2-specific IgG responses as compared to patients without cancer. Patients with hematologic cancers, however, had substantial defects in B cells and humoral immunity. These defects were associated with a high mortality or 45%, as compared to 25% in solid cancers. This difference in survival was driven by immune phenotype 4, which was characterized by robust CD4 T cell responses in conjunction with a diminished, but not absent CD8 T response. This phenotype (phenotype 4B), in the setting of preserved B cells seen in solid cancer patients, was associated with a low mortality of 9%. However, this phenotype in the setting of depleted B cells (phenotype 4A) seen in liquid cancer patients, was associated with a mortality of 61%. This highlights the fact that CD8 T cell responses that are normally sufficient may no longer be adequate in the setting of compromised humoral immunity. Thus, CD4 or B cells responses, in the absence of an intact CD8 T cell response, may not be sufficient to control an acute SARS-CoV-2 infection. This is reminiscent of published data demonstrating that uncoordinated immune responses in the elderly was associated with severe disease and poor outcomes^59^.

Finally, by leveraging a population of COVID-19 patients in the setting of B cell depletion (anti-CD20), we uncovered a critical protective role for CD8 T-cell responses. CD8 T cells are known to be critical for viral clearance, particularly in response to higher viral inocula^60^. Recent data from transgenic mouse models show that both CD4 and CD8 T cells are necessary for optimal viral clearance of SARS-CoV-2^61^. In patients treated with anti-CD20, absolute CD8 T cell count, but not CD4 counts, was associated with survival from COVID-19 and lower viral load. Although conclusions are limited by sample size, these data suggest that CD8 T cells play a key role in limiting SARS-CoV-2, even in the absence of humoral immunity. Indeed, SARS-CoV-2-specific CD8 T cell responses have been identified in acute and convalescent individuals^59, 62–65^. Further, in our cohort, absolute CD8 counts were predictive of outcomes in the broader cohort of patients with hematologic malignancy. The compensatory role of CD8 T cells was restricted to patients with hematologic, but not solid, malignancies. Thus, CD8 T cells likely play an important role in the setting of quantitative and qualitative B cell dysfunction in patients with lymphoma, multiple myeloma, and leukemia, undergoing anti-CD20, chemotherapy, or Bruton tyrosine kinase (BTK) inhibition. CD8 T cell counts may inform on the need for closer monitoring and a lower threshold for hospitalization in COVID-19 patients with hematologic malignancies. Furthermore, the clinical benefit of dexamethasone, which demonstrated an overall mortality benefit in hospitalized COVID-19+ patients but is known to suppress CD8 T cell responses^66^, should be investigated further in patients who recently received anti-CD20 therapy.

Recent analysis demonstrated that patients treated with B-cell depleting agents had the highest mortality rate, although this analysis did not account for whether the risk was modulated by CD8 count. Our findings do not exclude the possibility that B-cell depleting therapies may be associated with adverse outcomes in this population but rather extend these findings to suggest that an adequate CD8-dependent T cell response is essential for patients in whom humoral immunity is compromised. We did, however, observe a profound depletion of both naive CD4 and CD8 T cells in patients receiving B-cell depleting agents. Naive T-cells, and particularly naive CD4 T cells, are known to require tonic TCR signaling driven by APC-presented self-antigens for persistence^67, 68^. We speculate that depletion of functional B cells, particularly in the context of B cell depleting therapy, might lead to concomitant naive T cell depletion and a corresponding increase in the effector and activated CD8 T cells. Although the clinical relevance of naive T cell depletion in the setting of anti-CD20 is still unclear - it is notable that depletion of naive T cells in the elderly was associated with increased disease severity and poor outcomes^59^.

Importantly, both B-cell depleting therapies and cytotoxic chemotherapy agents which can compromise the T-cell compartment are mainstays of lymphoma therapy. Both are administered, often in combination, with curative intent for patients with aggressive lymphomas, but also for debulking or palliation in patients with indolent lymphomas. Based on our data, we would suggest that oncologists and patients considering treatment regimens that combine B cell depletion with cytotoxic agents carefully weigh the associated increased risk of immune dysregulation against the benefit of disease control when making an educated decision on whether to initiate such treatments, particularly in non-curative settings.

Finally, our finding that CD8 T cell immunity is critical for survival in hematologic malignancy patients with COVID-19 has profound implications for the vaccination of these patients. Both the Pfizer and Moderna vaccines, as well as the Johnson and Johnson vaccine currently under investigation, induce robust CD8 T cell responses in addition to humoral responses^69–71^. Our findings suggest that vaccination of hematologic patients might enhance the protective capacity of CD8 T-cells despite the likely absence of a humoral response. We are conducting ongoing studies to monitor the immune profile of patients undergoing vaccination prospectively to determine if this is the case. Ultimately, understanding how the immune response relates to disease severity, cancer type, and cancer treatment will provide important insight into the pathogenesis of and protective immunity from SARS-CoV-2, which may have implications for the development and prioritization of therapeutics and vaccines in cancer subpopulations.

## Methods:

### COVID-19 Outcomes in Patients with Cancer, COPE

#### General Design/Patient Selection

We conducted a prospective cohort study of patients with cancer hospitalized with COVID-19 (UPCC 06920). Informed consent was obtained from all patients. Adult patients with a current or prior diagnosis of cancer and hospitalized with a probable or confirmed diagnosis of COVID-19, as defined by the WHO criteria^72^, within the University of Pennsylvania Health System (UPHS) between April 28, 2020 and September 15, 2020 were approached for consent. Participating hospitals included the Hospital of the University of Pennsylvania, Presbyterian Hospital, Pennsylvania Hospital, and Lancaster General Hospital. The index date was defined as the first date of hospitalization within the health system for probable or confirmed COVID-19. Repeat hospitalizations within 7 days of discharge were considered within the index admission. Patients who died prior to being approached for consent were retrospectively enrolled. Patients were followed from the index date to 30-days following their discharge or until death by any cause. This study was approved by the institutional review boards of all participating sites.

#### Data Collection

Baseline characteristics including patient (age, gender, race/ethnicity, comorbidities, smoking history, body mass index) and cancer (tumor type, most recent treatment, ECOG performance status, active cancer status) factors as well as COVID-19 related clinical factors including change in levels of care, complications, treatments such as need for mechanical ventilation, laboratory values (complete blood counts with differentials and inflammatory markers including LDH, CRP, ferritin, and IL-6), and final disposition were extracted by trained research personnel using standardized abstraction protocols. Active cancer status was defined by diagnosis or treatment within 6 months of admission date. Cancer treatment status was determined by the most recent treatment within 3 months prior to admission date.

The primary study endpoint was all-cause mortality within 30-days of hospital discharge. Disease severity was categorized using the NIH ordinal scale including all post-hospitalization categories: 1,hospitalized, not requiring supplemental oxygen but requiring ongoing medical care; 2, hospitalized requiring any supplemental oxygen; 3, hospitalized requiring noninvasive mechanical ventilation or use of high-flow oxygen devices; 4, hospitalized receiving invasive mechanical ventilation or extracorporeal membrane oxygenation (ECMO); 5, death^73^, and was assessed every 7 days throughout a patients admission.

#### Statistical Analysis

Cohort characteristics were compared using standard descriptive statistics. One-time imputation of missing values for ECOG was done using the predicted mean value from an ordinal logistic model (proportional odds) of complete data. The ordinal model was fitted with forward stepwise selection, with entry at p=0.1 and removal at 0.2, using clinical variables expected to be correlated with ECOG performance status. Those variables included several items in the Charlson and severity score, and other clinical variables.

Univariate analyses examined demographic and clinical variables and cancer subtype (hematologic versus solid cancer) as predictors of death within 30 days of discharge and of ICU admission. Odds ratios and 95% CIs were used to generate the forest plot illustration. Baseline laboratory tests were compared by cancer type using Mann Whitney tests and available RT-PCR data was used to determine length of RT-PCR positivity by cancer type.

Rates of ICU admission and death were calculated for the overall cohort and stratified by cancer subtype. A multivariate logistic model was used to examine the adjusted effect of solid versus hematologic designation. Covariates included demographic variables of age and sex (race was omitted for missing data). Covariates also included clinical variables that attained a p-value of 0.1 in the univariate analyses. The final model included age, sex, smoking status, active disease status, and ECOG performance status. A cox proportional hazards regression model was also performed to determine the association between cancer type and mortality and identically adjusted for age, sex, smoking status, active cancer status, and ECOG performance status. Overall survival (OS) was measured from date of hospitalization to last follow up or death and the median OS was estimated using Kaplan-Meier method and differences by cancer subtype compared using log-rank test.

### Immune profiling of patients hospitalized for COVID-19, MESSI

Information on clinical cohort, sample processing, and flow cytometry is described in Mathew et al, Science 2020. Briefly, Patients admitted to the Hospital of the University of Pennsylvania with a positive SARS-CoV-2 PCR test were screened and approached for informed consent within 3 days of hospitalization. Peripheral blood was collected from all subjects and clinical data were abstracted from the electronic medical record into standardized case report forms. All participants or their surrogates provided informed consent in accordance with protocols approved by the regional ethical research boards and the Declaration of Helsinki. Methods for PBMC processing, flow cytometry, and antibodies used were previously described^15^.

### Serologic enzyme-linked immunosorbent assay (ELISA)

ELISAs were completed using plates coated with the receptor binding domain (RBD) of the SARS-CoV-2 spike protein as previously described^44^. Briefly, Prior to testing, plasma and serum samples were heat-inactivated at 56°C for 1 hour. Plates were read at an optical density (OD) of 450nm using the SpectraMax 190 microplate reader (Molecular Devices). Background OD values from the plates coated with PBS were subtracted from the OD values from plates coated with recombinant protein. Each plate included serial dilutions of the IgG monoclonal antibody CR3022, which is reactive to the SARS-CoV-2 spike protein, as a positive control to adjust for inter assay variability. Plasma and serum antibody concentrations were reported as arbitrary units relative to the CR3022 monoclonal antibody. A cutoff of 0.48 arbitrary units was established from a 2019 cohort of pre-pandemic individuals and used for defining seropositivity.

### Flow Cytometry and statistical analysis

Samples were acquired on a 5 laser BD FACS Symphony A5. Standardized SPHERO rainbow beads (Spherotech, Cat#RFP-30–5A) were used to track and adjust PMTs over time. UltraComp eBeads (ThermoFisher, Cat#01–2222-42) were used for compensation. Up to 2 × 10^6 live PBMC were acquired per each sample. During the early sample acquisition period, three antibodies in the flow panel were changed. Three cancer patients and twelve non-cancer patients were stained using this earlier flow panel. Flow features of these patients were visually assessed for batch variations against data from the later flow panel. The three cancer patients were included with the rest of the cohort when batch effects were determined to have little impact on confidence in gated populations. These three cancer patients were excluded in analysis of cell populations defined by proteins associated with the three changed antibodies.

Due to the heterogeneity of clinical and flow cytometric data, non-parametric tests of association were preferentially used throughout the study. Tests of association between mixed continuous variables versus non-ordered categorical variables (n=2) were performed by MannWhitney test. Tests of association between binary variables versus non-ordered categorical variables (n=2) were performed using Pearson Chi Square test. All tests were performed using a nominal significance threshold of P<0.05 with Prism version 9 (GraphPad Software) and Excel (Microsoft Office Suite). Classification and Regression Tree analysis (CART) was performed using R package ‘rpart’.

### High dimensional data analysis of flow cytometry data

UMAP analyses were conducted using R package *uwot*. FlowSOM analyses were performed on Cytobank (https://cytobank.org). Lymphocytes and non-naive CD8 T cells were analyzed separately. An artifact due to monocyte contamination was removed from the FCS as defined by high CD16 and side scatter area (SSC-A). UMAP analysis was performed using equal down sampling of 10000 cells from each FCS file in lymphocytes and 1500 cells in non-naive CD8 T cells, with a nearest neighbors of 15, minimum distance of 0.01, number of components of 2, and a euclidean metric. The FCS files were then fed into the FlowSOM clustering algorithm. A new self-organizing map (SOM) was generated for both lymphocytes and non-naive CD8 using hierarchical consensus clustering. For each SOM, 225 clusters and 10 metaclusters were identified. For lymphocytes, the following markers were used in the UMAP and FlowSOM analysis: CD45RA, PD-1, IgD, CXCR5, CD8, CD19, CD3, CD16, CD138, Eomes, TCF-1, CD38, CD95, CCR7, CD21, Ki-67, CD27, CD4, CX3CR1, CD39, T-bet, HLA-DR, and CD20. For non-naive CD8 T cells, the following markers were used: CD45RA, PD-1, CXCR5, CD16, Eomes, TCF-1, CD38, CCR7, Ki-67, CD27, CX3CR1, CD39, T-bet, and HLA-DR. For FlowSOM analysis of non-naive CD8 T cells, two patients at day seven without data from day zero were included. Heatmaps were visualized using R function *pheatmap*.

To group individuals based on lymphocyte landscape, pairwise Earth Mover’s Distance (EMD) value was calculated on the lymphocyte UMAP axes using the *emdist* package in R. Resulting scores were hierarchically clustered using the *hclust* package in R.

### Immune profiling of patients hospitalized for COVID-19, MSKCC

Patients admitted to Memorial Sloan Kettering Cancer Center with a positive SARS-CoV-2 PCR test were eligible for inclusion. For inpatients, clinical data were abstracted from the electronic medical record into standardized case report forms. Clinical laboratory data were abstracted from the date closest to research blood collection. Peripheral blood was collected into BD Horizon Dri tubes (BD, Cat#625642). Immunophenotyping of peripheral blood mononuclear cells via flow cytometry was performed in the MSKCC clinical laboratory. The lymphocyte panel included CD45 FITC (BD, 340664, clone 2D1), CD56+16 PE (BD 340705, clone B73.1; BD 340724, clone NCAM 16.2), CD4 PerCP-Cy5.5 (BD 341653, clone SK3), CD45RA PC7 (BD 649457, clone L48), CD19 APC (BD 340722, clone SJ25C1), CD8 APC-H7 (BD 641409, clone SK1), and CD3 BV 421 (BD 562426, clone UCHT1). The naive/effector T panel included CD45 FITC (BD 340664, clone 2D1), CCR7 PE (BD 560765, clone 150503), CD4 PerCP-Cy5.5 (BD 341653, clone SK3), CD38 APC (BioLegend, 303510, clone HIT2), HLA-DR V500 (BD 561224, clone G46–6), CD45RA PC7 (BD 649457, clone L48), CD8 APC-H7 (BD 641409, clone SK1), and CD3 BV 421 (BD 562426, clone UCHT1). The immune phenotypes were based on NIH vaccine consensus panels and the Human Immunology Project^74^. Samples were acquired on a BD Facs Canto using FACSDiva software.

### Data Availability Statement:

Flow Cytometry data collected in this study was deposited to the Human Pancreas Analysis Program (HPAP-RRID:SCR_016202) Database and Cytobank62

## Extended Data

## Supplementary Material

1

## Figures and Tables

**Table 1 |:** COPE: Patient demographics and clinical characteristics.

	Total (N=100)
**Age, median (IQR)**	68 (57.5–77.5)
**Gender, female**	48 (48%)
**Race**	
Black	54 (54%)
White	33 (33%)
Asian	4 (4%)
Hispanic	3 (3%)
Unknown	6 (6%)
**Smoking History, Ever**^+^	57 (57%)
**Comorbidities**	
Cardiac	78 (78%)
Pulmonary	41 (41%)
**Use of immunosuppressive drugs**^++^	30 (30%)
**BMI, median (IQR)**	26.84 (23.2–31.5)
**Cancer Type**	
Solid malignancy	78 (78%)
Genitourinary	19 (19%)
Breast	14 (14%)
Gastrointestinal	14 (14%)
Thoracic	9 (9%)
Other^+++^	8 (8%)
Gynecologic	7 (7%)
Head and Neck	4 (4%)
Sarcoma	3 (3%)
Heme malignancy	22 (22%)
Lymphoma	10 (10%)
Leukemia	7 (7%)
Myeloma	3 (3%)
MDS/MPN	2 (2%)
**Cancer Status, Active**^#^	46 (46%)
**Cancer treatment in last 3 months**	
Active surveillance/surgery	53 (53%)
Cytotoxic Chemotherapy	24 (24%)
Hormone therapy	15 (15%)
Other*	8 (8%)
**ECOG Performance Status**	N=73
0–1	37 (50.7%)
2	13 (17.8%)
3–4	23 (31.5%)

+ Current or prior smoker

++ Exposure to immunosuppressive medications not including cancer treatment

+++ Tumor types with less than 2 subjects: CNS-2, Thyroid-2, Thymus-1, Neuroendocrine-1

# Diagnosis or treatment within 6 months

* Single agent immunotherapy, targeted therapy, monoclonal antibodies

**Table 2 |:** COPE: COVID-19 related treatment and outcomes.

	Total (N=100)	Solid (N=78)	Heme (N=22)
**COVID-19 Disease Severity**			
At Presentation			
No Supplemental Oxygen	35 (35.0%)	28 (35.9%)	7 (31.85)
Supplemental Oxygen	44 (44.0%)	32 (41.0%)	12 (54.6%)
Non-invasive ventilation	9 (9.00%)	7 (8.97%)	2 (9.09%)
Invasive ventilation	12 (12.0%)	11 (14.1%)	1 (4.55%)
Maximum throughout hospitalization			
No supplemental Oxygen	28 (28.0%)	24 (30.8%)	4 (18.2%)
Supplemental Oxygen	24 (24.0%)	19 (24.4%)	4 (18.2%)
Non-invasive ventilation	11 (11.0%)	8 (10.3%)	3 (13.6%)
Invasive ventilation	9 (9.00%)	9 (11.5%)	0 (0.00%)
Death	28 (28.0%)	18 (23.1%)	12 (54.5%)
**COVID-19 Directed Treatment**			
Steroids	51 (51.0%)	39 (50.0%)	12 (54.6%)
Remdesivir	18 (18.0%)	13 (16.7%)	5 (22.7%)
Convalescent Plasma	10 (10.0%)	6 (7.69%)	4 (18.2%)
**COVID-19 Outcomes**			
Thrombosis	11 (11.0%)	7 (9.09%)	4 (18.2%)
Intubation	28 (28.0%)	21 (26.9%)	7 (31.8%)
ICU admission	48 (48.0%)	37 (47.4%)	11 (50.0%)
Death	38 (38.0%)	26 (33.3%)	12 (54.6%)
Hospital Length of stay, median (IQR)	8 (4–19)	8 (4–20)	8 (4–18)

**Table 3 |:** COPE: Event rates and point estimates of outcomes by cancer type.

	Heme	Solid
**Death within 30 days of discharge**		
Event rate (%)	12 (54.6%)	26 (33.3%)
Unadjusted OR (95% CI)	2.4 (0.82–7.06)	ref
Adjusted OR (95% CI)^+^	3.3 (1.01–10.8)	ref
Adjusted HR (95% CI)^+^	2.6 (1.19–5.54)	ref

+ Logistic regression computed odds ratio (OR) and Cox regression computed hazard ratio (HR), respectively. Adjusted for age, gender, smoking status, active cancer status, and ECOG performance status.
