# Supplementary material for: CD8 T cells compensate for impaired humoral immunity in COVID-19 patients with hematologic cancer
Source: Res Sq. 2021 Feb 2:rs.3.rs-162289. Preprint. [Version 1] doi: 10.21203/rs.3.rs-162289/v1 (PMC7872363; doi:10.21203/rs.3.rs-162289/v1)
Supplement: 1 [file NIHPPrs162289v1-supplement-1.pdf]

Supplementary Table 1 | COPE: Patient demographics and clinical characteristics by tumor type.

	Solid (N=78)	Heme (N=22)
Age, median (IQR)	70.5 (57-78)	64.5 (60-77)
Gender, female	39 (50.0%)	9 (40.9%)
Race		
White	24 (30.8%)	9 (40.9%)
Non-white	49 (62.8%)	12 (54.6%)
Unknown	5 (6.41%)	1 (4.55%)
Smoking History, Current/Prior	44 (56.4%)	13 (59.1%)
Comorbidities		
Cardiac	63 (80.8%)	15 (68.2%)
Pulmonary	38 (48.7%)	3 (13.6%)
Use of immunosuppressive drugs[+]	23 (29.5%)	7 (31.8%)
BMI, median (IQR)	26.6 (23.2-30.9)	28.7 (24.0-33.4)
Cancer Status, Active[++]	32 (41.0%)	14 (63.6%)
Cancer Treatment		
Treatment in last 3 months		
Cytotoxic Chemotherapy	16 (20.5%)	8 (36.4%)
Hormone therapy	15 (19.2%)	0 (0.00%)
Active Surveillance/surgery	43 (55.1%)	10 (45.5%)
Other*	4 (5.13%)	4 (18.2%)
ECOG Performance Status	N=58	N=15
0-1	28 (48.3%)	9 (60.0%)
2	9 (15.5%)	4 (26.7%)
3-4	21 (36.2%)	2 (13.3%)

*Single agent immunotherapy, targeted therapy, monoclonal antibodies
[+]Exposure to immunosuppressive medications not including cancer treatment
[++]Diagnosis or treatment within 6 months

Supplementary Table 2 | MESSI: Patient demographics and clinical characteristics by cancer status.

	Non-Cancer (N=108)	Cancer (N=22)	Overall (N=130)
Gender			
Female	49 (45.4%)	14 (63.6%)	63 (48.5%)
Male	59 (54.6%)	8 (36.4%)	67 (51.5%)
Age (median)	60	66	60.5
Race			
Asian	5 (4.6%)	1 (4.5%)	6 (4.6%)
Black	74 (68.5%)	16 (72.7%)	90 (69.2%)
White	28 (25.9%)	5 (22.7%)	33 (25.4%)
Pacific Islander	1 (0.9%)	0 (0%)	1 (0.8%)
Symptoms (Days before hospitalization) (median)	9	8	9
Severity (At hospitalization) (median)	3.5	3	3
Mortality (28 days)	12 (11.1%)	8 (36.4%)	20 (15.4%)

Supplementary Table 3 | MESSI: Patient demographics and clinical characteristics by cancer type.

	Heme (N=7)	Solid (N=15)	Overall (N=22)
Gender			
Female	4 (57.1%)	10 (66.7%)	14 (63.6%)
Male	3 (42.9%)	5 (33.3%)	8 (36.4%)
Age (median)	67	65	66
Race			
Asian	0 (0%)	1 (6.7%)	1 (4.5%)
Black	5 (71.4%)	11 (73.3%)	16 (72.7%)
White	2 (28.6%)	3 (20.0%)	5 (22.7%)
Symptoms (Days before hospitalization) (median)	9	7.5	8
Severity (At hospitalization) (median)	3	4	3
Mortality (28 days)	2 (28.6%)	6 (40.0%)	8 (36.4%)
COVID Treatments			
Remdesivir	1 (14.3%)	2 (13.3%)	3 (13.6%)
Convalescent Plasma	1 (14.3%)	3 (20.0%)	4 (18.2%)
Early Steroids	4 (57.1%)	5 (33.3%)	9 (40.9%)

Supplementary Table 4 | MESSI: Cancer type and cancer treatment.

	Heme (N=7)	Solid (N=15)	Overall (N=22)
Cancer Type			
CML	1 (14.3%)	0 (0%)	1 (4.5%)
CTCL	1 (14.3%)	0 (0%)	1 (4.5%)
Lymphoma	1 (14.3%)	0 (0%)	1 (4.5%)
Mantle Cell Lymphoma	1 (14.3%)	0 (0%)	1 (4.5%)
MM	1 (14.3%)	0 (0%)	1 (4.5%)
MPN	1 (14.3%)	0 (0%)	1 (4.5%)
Myeloma	1 (14.3%)	0 (0%)	1 (4.5%)
Bladder	0 (0%)	1 (6.7%)	1 (4.5%)
Breast	0 (0%)	6 (40.0%)	6 (27.3%)
GBM	0 (0%)	1 (6.7%)	1 (4.5%)
Head and Neck	0 (0%)	1 (6.7%)	1 (4.5%)
NSCLC	0 (0%)	1 (6.7%)	1 (4.5%)
Ovarian	0 (0%)	1 (6.7%)	1 (4.5%)
Pancreas	0 (0%)	1 (6.7%)	1 (4.5%)
Prostate	0 (0%)	2 (13.3%)	2 (9.1%)
SCLC	0 (0%)	1 (6.7%)	1 (4.5%)
Cancer Treatment			
αCD20 + Chemotherapy	1 (14.3%)	0 (0%)	1 (4.5%)
Chemo	5 (71.4%)	4 (26.7%)	9 (40.9%)
None	1 (14.3%)	1 (6.7%)	2 (9.1%)
Chemotherapy + Radiation	0 (0%)	1 (6.7%)	1 (4.5%)
Hormonal	0 (0%)	4 (26.7%)	4 (18.2%)
Hormonal + CDK Inhibitor	0 (0%)	1 (6.7%)	1 (4.5%)
Hormonal + Radiation	0 (0%)	1 (6.7%)	1 (4.5%)
ICB	0 (0%)	3 (20.0%)	3 (13.6%)

Supplementary Table 5 | MSKCC: Patient demographics and clinical characteristics by cancer type.

	Heme (N=45)	Solid (N=39)	Overall (N=84)
Gender			
Female	21 (46.7%)	20 (51.3%)	41 (48.8%)
Male	24 (53.3%)	19 (48.7%)	43 (51.2%)
Age (median)	64	66	65
Race			
Asian	2 (4.4%)	3 (7.7%)	5 (6.0%)
Black	5 (11.1%)	4 (10.3%)	9 (10.7%)
White	38 (84.4%)	30 (76.9%)	68 (81.0%)
Disease Severity (median)	4	3	4
Mortality (In hospital)	20 (44.4%)	8 (20.5%)	28 (33.3%)
COVID Treatments			
Remdesivir	12 (26.7%)	6 (15.4%)	18 (21.4%)
Convalescent Plasma	25 (55.6%)	14 (35.9%)	39 (46.4%)
Early Steroids	17 (37.8%)	21 (53.8%)	38 (45.2%)

Supplementary Table 6 | MSKCC: Cancer type and cancer treatment.

	Heme (N=45)	Solid (N=39)	Overall (N=84)
Cancer Type			
ALL	4 (8.9%)	0 (0%)	4 (4.8%)
AML	6 (13.3%)	0 (0%)	6 (7.1%)
CLL	4 (8.9%)	0 (0%)	4 (4.8%)
Lymphoma	23 (51.1%)	0 (0%)	23 (27.4%)
MDS/Myelofibrosis	3 (6.7%)	0 (0%)	3 (3.6%)
Myeloma	5 (11.1%)	0 (0%)	5 (6.0%)
Bladder	0 (0%)	2 (5.1%)	2 (2.4%)
Breast	0 (0%)	8 (20.5%)	8 (9.5%)
CNS	0 (0%)	3 (7.7%)	3 (3.6%)
Colorectal	0 (0%)	5 (12.8%)	5 (6.0%)
GYN	0 (0%)	3 (7.7%)	3 (3.6%)
Head and Neck	0 (0%)	1 (2.6%)	1 (1.2%)
Kidney	0 (0%)	1 (2.6%)	1 (1.2%)
Liver	0 (0%)	1 (2.6%)	1 (1.2%)
Lung	0 (0%)	5 (12.8%)	5 (6.0%)
Melanoma	0 (0%)	2 (5.1%)	2 (2.4%)
Prostate	0 (0%)	3 (7.7%)	3 (3.6%)
Renal	0 (0%)	1 (2.6%)	1 (1.2%)
Sarcoma	0 (0%)	2 (5.1%)	2 (2.4%)
Thymoma	0 (0%)	1 (2.6%)	1 (1.2%)
Thyroid	0 (0%)	1 (2.6%)	1 (1.2%)
Cancer Treatment			
αCD20	9 (20.0%)	0 (0%)	9 (10.7%)
αCD20 + chemo	9 (20.0%)	0 (0%)	9 (10.7%)
αnti-CD30	1 (2.2%)	0 (0%)	1 (1.2%)
αHER2	0 (0%)	2 (5.1%)	2 (2.4%)
AXL inhibitor	1 (2.2%)	0 (0%)	1 (1.2%)
Bispecific	1 (2.2%)	0 (0%)	1 (1.2%)
BTK inhibitor	4 (8.9%)	0 (0%)	4 (4.8%)
CAR-T	1 (2.2%)	0 (0%)	1 (1.2%)
Chemotherapy	8 (17.8%)	15 (38.5%)	23 (27.4%)
EZH inhibitor	1 (2.2%)	0 (0%)	1 (1.2%)
PI3K Inhibitor	1 (2.2%)	0 (0%)	1 (1.2%)
Proteasome inhibitor	3 (6.7%)	0 (0%)	3 (3.6%)
Radiation	1 (2.2%)	0 (0%)	1 (1.2%)
Tyrosine kinase inhibitor	1 (2.2%)	0 (0%)	1 (1.2%)
Hormonal	0 (0%)	5 (12.8%)	5 (6.0%)
Immune checkpoint blockade	0 (0%)	7 (17.9%)	7 (8.3%)
VEGF inhibitor	0 (0%)	1 (2.6%)	1 (1.2%)
None	4 (8.9%)	9 (23.1%)	13 (15.5%)
